# Sphingosine kinase 1-associated autophagy differs between neurons and astrocytes

**DOI:** 10.1038/s41419-018-0599-5

**Published:** 2018-05-09

**Authors:** Jose F. Moruno-Manchon, Ndidi-Ese Uzor, Chandrashekar R. Ambati, Vivekananda Shetty, Nagireddy Putluri, Chinnaswamy Jagannath, Louise D. McCullough, Andrey S. Tsvetkov

**Affiliations:** 1 Department of Neurobiology and Anatomy, The University of Texas McGovern Medical School, Houston, TX 77030 USA; 2 The University of Texas Graduate School of Biomedical Sciences, Houston, TX 77030 USA; 3 Department of Molecular and Cellular Biology, Baylor College of Medicine, Houston, TX 77030 USA; 4 Department of Pathology and Laboratory Medicine, The University of Texas McGovern Medical School, Houston, TX 77030 USA; 5 Department of Neurology, The University of Texas McGovern Medical School at Houston, Houston, TX 77030 USA; 6 UT Health Consortium on Aging, The University of Texas McGovern Medical School, Houston, TX 77030 USA

## Abstract

Autophagy is a degradative pathway for removing aggregated proteins, damaged organelles, and parasites. Evidence indicates that autophagic pathways differ between cell types. In neurons, autophagy plays a homeostatic role, compared to a survival mechanism employed by starving non-neuronal cells. We investigated if sphingosine kinase 1 (SK1)-associated autophagy differs between two symbiotic brain cell types—neurons and astrocytes. SK1 synthesizes sphingosine-1-phosphate, which regulates autophagy in non-neuronal cells and in neurons. We found that benzoxazine autophagy inducers upregulate SK1 and neuroprotective autophagy in neurons, but not in astrocytes. Starvation enhances SK1-associated autophagy in astrocytes, but not in neurons. In astrocytes, SK1 is cytoprotective and promotes the degradation of an autophagy substrate, mutant huntingtin, the protein that causes Huntington’s disease. Overexpressed SK1 is unexpectedly toxic to neurons, and its toxicity localizes to the neuronal soma, demonstrating an intricate relationship between the localization of SK1’s activity and neurotoxicity. Our results underscore the importance of cell type-specific autophagic differences in any efforts to target autophagy therapeutically.

## Introduction

Autophagy is a cellular process essential for removing aggregated proteins and damaged organelles from cells^1^. Autophagy has been extensively characterized in yeast cells and mammalian cell lines, but not in neurons. Mechanisms of autophagy may differ between neuronal and other cells. Starvation, the best-known inducer of autophagy in most cells,  does not induce autophagy in cortical neurons of starving mice^2^. Starvation does not effectively induce autophagy in cultured neurons^3^. Starvation downregulates mTORC1 signaling; however, autophagy is not affected^3^. Regulation of autophagy is different between neuronal compartments and more complex than thought^3–5^.

Sphingosine kinases (SK) catalyze the phosphorylation of sphingosine to form sphingosine-1-phosphate (S1P). Mammalian cells contain two sphingosine kinases: cytoplasmic SK1 and nuclear/mitochondrial SK2. S1P phosphatases remove the phosphate group from S1P, generating sphingosine^6^. S1P lyase (S1PL) irreversibly degrades S1P to phosphoethanolamine and hexadecenal^7^. Increasing endogenous S1P levels by overexpressing SK1 enhances formation of autophagosomes and stimulates cytoprotective autophagy in cancerous cell lines^8, 9^. In fibroblasts, SK1 regulates autophagy and endosomal trafficking^10, 11^. S1PL in *Legionella pneumophila* inhibits autophagy^12^. Intriguingly, in neurons, S1PL positively modulates autophagy by generating phosphatidylethanolamine from S1P^13^. S1P-phosphatase regulates autophagy, but its downregulation leads to increased cleavage of the Atg5 protein, which indicates the activation of apoptosis^14^. Thus, the precise role of the S1P pathway in autophagy and whether it is always beneficial or can be cytotoxic, or even both—is not resolved.

Here, we investigated whether there is a difference in SK1-associated autophagy between neurons and astrocytes—two symbiotic brain cell types. We discovered that stimulating autophagy with benzoxazines upregulates SK1 and autophagy in neurons, but not in astrocytes. Amino acid withdrawal stimulates SK1-associated autophagy in astrocytes, but not in neurons. Although benzoxazines stimulate neuroprotective autophagy and upregulate SK1, overexpressed SK1 is paradoxically toxic to neurons. We used a new technology that, in living neurons, allows us to analyze neuronal features and measure longevity of the same neurons. Active SK1 localized to the soma was associated with neuronal death,while neurons that contained active SK1 in their neurites survived better. Our data highlight the significance of cell type-specific and compartment-dependent differences in autophagy to target autophagy therapeutically.

## Results

### Induction of SK1-associated autophagy is different in neurons and astrocytes

Autophagy has been characterized mostly in yeast and mammalian non-neuronal cells. Autophagy in astrocytes is also poorly studied. Earlier, we showed that SK1-associated autophagy is different between neurons and neuron-like neuroblastoma SH-SY5Y cells^8^. Here, we hypothesized that SK1 and the S1P pathway regulate autophagy differently in neurons and astrocytes.

**Fig. 1:**
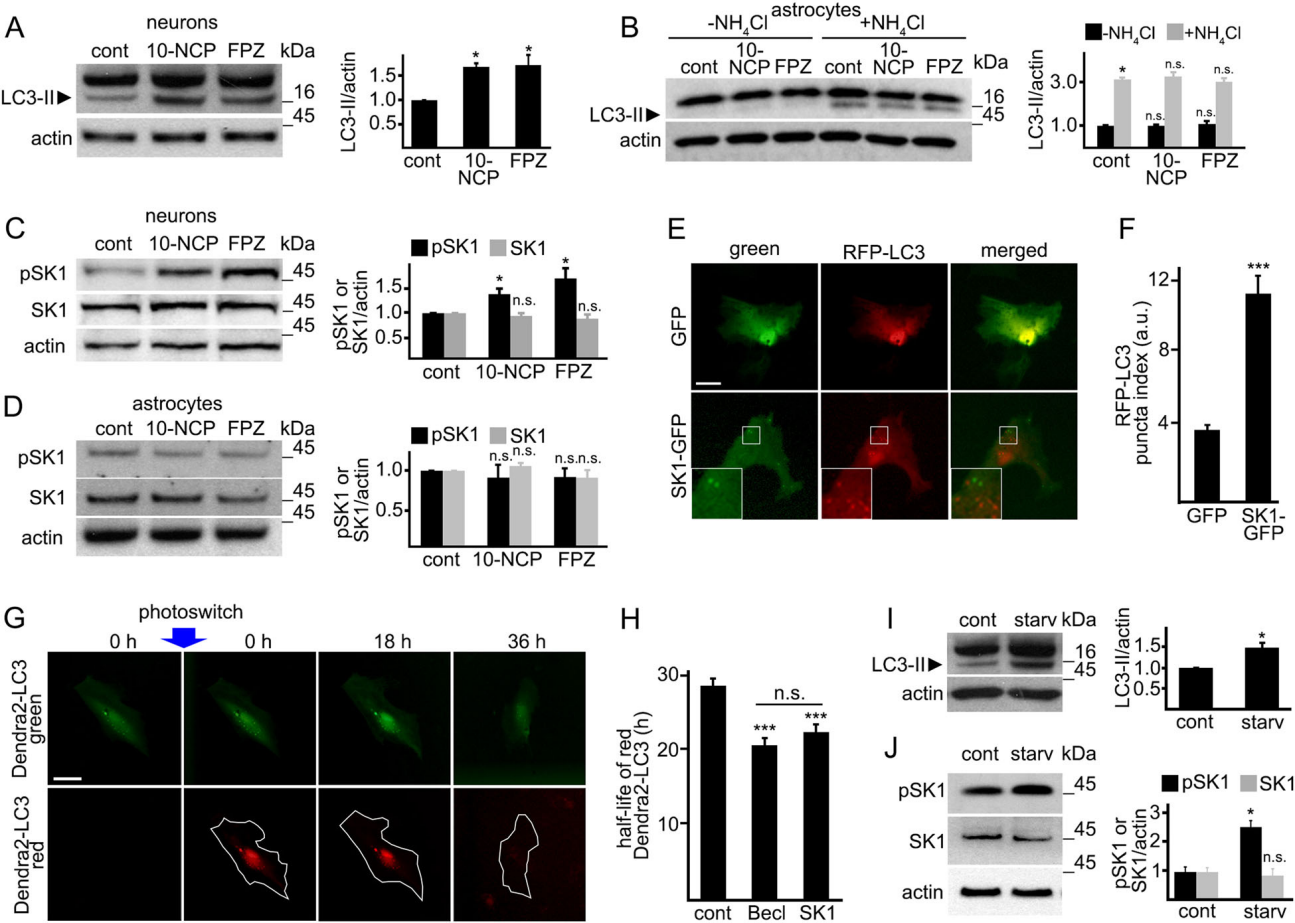
SK1-associated autophagy differs between neurons and astrocytes. **a** Cultured primary cortical neurons were treated with a vehicle (control, cont), or with 10-NCP (10-NCP, 5 µM), or fluphenazine (FPZ, 5 µM) for 4 h. Lysates were analyzed by western blotting with antibodies against LC3. Actin was used as a loading control. Bar graphs represent the quantification of the LC3-II intensities normalized to actin. *p (cont vs 10-NCP) = 0.042, *p (cont vs FPZ) = 0.024. **b** Cultured primary astrocytes were treated with a vehicle (control, cont), or with 10-NCP (10-NCP, 5 µM), or fluphenazine (FPZ, 5 µM), with or without NH_4_Cl for 4 h. Lysates were analyzed by western blotting with antibodies against LC3. Actin was used as a loading control. Bar graphs represent the quantification of the LC3-II intensities normalized to actin. *p* LC3 (cont vs 10-NCP) = 0.0749, *p* LC3 (cont vs FPZ) = 0.5297 (one-way ANOVA). ***p (cont vs cont + NH_4_Cl) = 0.0002 (*t*-student), *p* (cont + NH_4_Cl vs 10-NCP + NH_4_Cl) = 0.7699, *p* (cont + NH_4_Cl vs FPZ + NH_4_Cl) = 0.2257 (one-way ANOVA). Results were pooled from three independent experiments. **c**, **d** Cultured primary cortical neurons (**c**) and primary astrocytes (**d**) were treated with a vehicle (control, cont) or with 10-NCP (5 µM), or fluphenazine (FPZ, 5 µM) for 4 h. Lysates were analyzed by western blotting with antibodies against phosphorylated SK1 (pSK1) or pan-SK1 (SK1). Actin was used as a loading control. Bar graphs represent the quantification of the pSK1 and SK1 band intensities normalized to actin. In neurons: *p pSK1 (cont vs 10-NCP) = 0.0269, *p pSK1 (cont vs FPZ) = 0.0224; *p* SK1 (cont vs 10-NCP) = 0.7939, *p* SK1 (cont vs FPZ) = 0.208 (one-way ANOVA). In astrocytes: *p* pSK1 (cont vs 10-NCP) = 0.05356, *p* pSK1 (cont vs FPZ) = 0.9909; *p* SK1 (cont vs 10-NCP) = 0.1158, *p* SK1 (cont vs FPZ) = 0.4113 (one-way ANOVA). Results were pooled from three independent experiments. **e** Primary astrocytes were transfected with GFP and RFP-LC3 or with SK1-GFP and RFP-LC3. Twenty-four hours after transfection, astrocytes were imaged. Scale bar is 10 µm. A part of the image is zoomed in to visualize the SK1-GFP and RFP-LC3 puncta. **f** Quantification of the RFP-LC3 puncta index from **e**. ***p (GFP vs SK1-GFP) = 0.0001 (*t* test). One hundred and fifty cells were analyzed from three independent experiments. **g** The photoswitchable protein Dendra2 targeted to LC3 as a surrogate for the flux through autophagy. Brief irradiation with short-wave-length visible light causes Dendra2-LC3 to undergo an irreversible conformational change (“photoswitch”) and emit red fluorescence that can be tracked until altered Dendra2-LC3 is cleared. A cell is outlined to show cellular morphology. Scale bar is 10 µm. **h** Primary astrocytes were transfected with Dendra2-LC3 and an empty plasmid or with Dendra2-LC3 and a plasmid that encodes Beclin1, or Dendra2-LC3 and SK1. After the photoswitch, astrocytes were longitudinally imaged. The change in red fluorescence intensity over time was used to calculate the half-life of Dendra2-LC3. The single-cell half-life of Dendra2-LC3 was reduced by SK1 expression. Beclin1 was used as a positive control^16^. Fifty astrocytes were analyzed per condition from two independent experiments. ***p (control vs Becl) = 0.0001, ***p (control vs SK1) = 0.0001; *p* (Becl vs SK1) = 0.084 (one-way ANOVA). **i**, **j** Cultured primary astrocytes were maintained in DMEM supplemented with 10% fetal bovine serum (control, cont) or in Hanks’ balanced salt solution (starvation, starv) for 4 h. Samples were also treated with 10 mM NH_4_Cl to block the last step of autophagy-mediated degradation. Lysates were analyzed by western blotting with antibodies against LC3 (**i**) or with antibodies against phosphorylated SK1 (pSK1) or pan-SK1 (SK1) (**j**). Actin was used as a loading control. Bar graphs represent the quantification of the LC3-II, pSK1, or SK1 intensities normalized to actin. *p LC3 (control vs starv) = 0.017; *p* pSK1 (control vs starv) = 0.0072, *p* SK1 (control vs starv) = 0.331 (one-way ANOVA). Results were pooled from three independent experiments. n.s. not significant

Previously, we discovered a series of small molecules that induce autophagy in primary neurons^15^. Two prototypic benzoxazine compounds from this family of autophagy inducers, 10-NCP and fluphenazine (FPZ), promote protective neuronal autophagy and protect neurons from misfolded proteins^15, 16^. In primary cortical neurons, 10-NCP and FPZ increased LC3-II levels (Fig. 1a), confirming our previous reports^16, 17^. To test if 10-NCP and FPZ alter autophagy in astrocytes, primary astrocytes were treated with 10-NCP and FPZ, with or without NH_4_Cl, which neutralizes the lysosomal pH and blocks autophagic degradation (Fig. 1b). Neither upregulated astrocytic autophagy.

We reported that, in neurons, 10-NCP and FPZ induce relocalization of SK1-GFP into endosomes and amphisomes^17^. Here, we tested if 10-NCP and FPZ upregulate SK1 in astrocytes and compared these data to neurons. Neurons and astrocytes were treated with a vehicle or with 10-NCP or FPZ. 10-NCP and FPZ promoted phosphorylation of endogenous SK1 in neurons (Fig. 1c). They did not affect levels of phospho-SK1 in astrocytes (Fig. 1d). Thus, SK1-associated autophagy differs in neurons and astrocytes.

Does SK1 regulate autophagy in astrocytes at all? We expressed an autophagy marker LC3 fused to а monomeric fluorescent protein TagRFP (RFP-LC3 thereafter), along with SK1-GFP or GFP (green fluorescent protein) in primary astrocytes. Two astrocytic cohorts were analyzed. SK1-GFP-expressing astrocytes contained more RFP-LC3-positive autophagosomes than GFP-expressing cells, indicating that SK1 upregulates astrocytic autophagy (Fig. 1e, f).

To determine if SK1 affects the autophagic flux in astrocytes, we used an optical pulse-chase method based on a photoswitchable protein, Dendra2. Brief irradiation with short-wavelength visible light irreversibly changes the conformation of Dendra2 (“photoswitch”), which emits green fluorescence. Photoswitched Dendra2 emits red fluorescence. The Dendra2-based optical technique has been used to study autophagy^8, 16–18^, protein degradation^16, 18, 19^, the dynamics of synaptic proteins^20^, and mitochondrial dynamics^21^. To determine if SK1 changes autophagic flux, astrocytes were transfected with (1) Dendra2-LC3 and an empty plasmid, or (2) Dendra2-LC3 and a plasmid encoding Beclin1 that enhances autophagy (a positive control), or (3) Dendra2-LC3 and a plasmid encoding SK1. Transfected astrocytes were photoswitched, and the decay of red Dendra2 fluorescence was measured over time. We discovered that astrocytes overexpressing SK1 and Beclin1 had enhanced degradation of photoswitched Dendra2-LC3 (Fig. 1g, h). These data demonstrate that SK1 also regulates autophagy in astrocytes; however, pathways that activate SK1 may depend on cell type.

We then tested if starvation would upregulate SK1-associated autophagy in astrocytes. First, primary astrocytic cultures were incubated in amino acid-free medium, and the levels of an autophagy marker LC3-II were measured by western blotting. Withdrawing essential amino acids promoted LC3-II accumulation and astrocytic autophagy (Fig. 1i). Second, astrocytes were incubated in amino acid-free media, and levels of phospho-SK1 were measured. Unlike 10-NCP and FPZ, starvation enhanced SK1 phosphorylation in astrocytes (Fig. 1j). These findings indicate SK1 modulates autophagy in neurons and astrocytes, but pathways that govern SK1-associated autophagy in these cell types differ.

### Overexpressed SK1 protects astrocytes during starvation

**Fig. 2:**
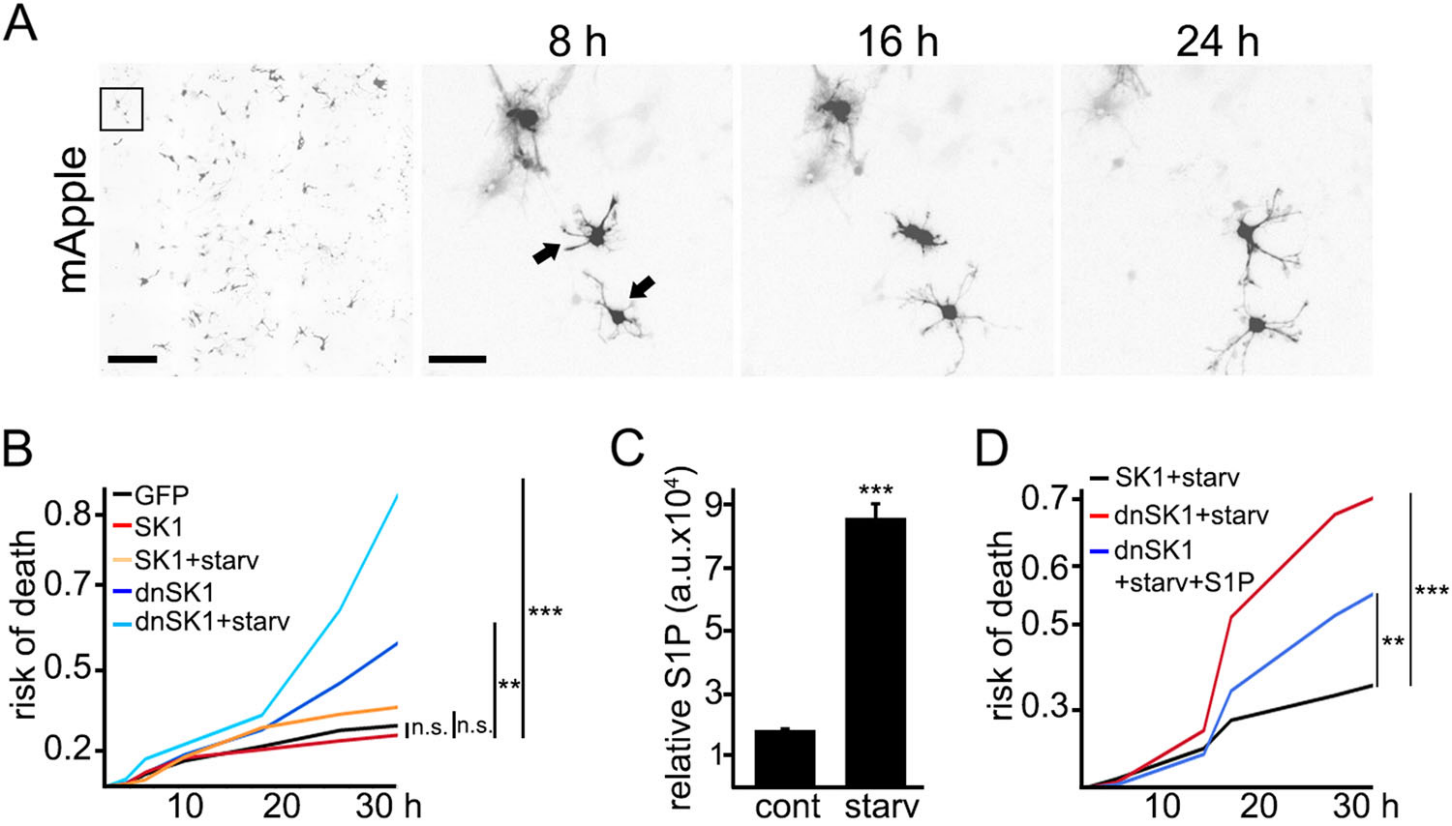
Overexpressed SK1 protects astrocytes during starvation. **a** An example of longitudinal imaging of astrocytes. Primary astrocytes were transfected with mApple to visualize their morphology. After transfection, the same group of astrocytes were imaged longitudinally with an automated microscope at different time points. The first image is a montage of non-overlapping images captured in one well of a 24-well plate. Scale bar is 400 µm. The adjacent panels are zoomed in to three cells, to demonstrate longitudinal single-cell tracking. Scale bar is 50 µm. Arrows indicate two astrocytes that died before the last imaging event. **b** Astrocytes were transfected with mApple and GFP, as control, or with mApple and SK1-GFP, or with mApple and a plasmid that encodes a dominant-negative form of SK1 (dnSK1) tagged to GFP. SK1- or dnSK1-expressing cells were maintained in DMEM supplemented with 10% fetal bovine serum (SK1 or dnSK1) or in Hanks’ balanced salt solution (starvation, starv; SK1 + starv or dnSK1 + starv) and tracked with an automated microscope for 36 h. Risk of death curves demonstrate that SK1 expression protects astrocytes during starvation. ***p (dnSK1 vs dnSK1 + starv) = 0.0001, **p (GFP vs dnSK1) = 0.0253; *p* (GFP vs SK1) = 0.495, *p* (SK1 vs SK1 + starv) = 0.2184 (log-rank test). Fifty astrocytes per group were analyzed from three independent experiments. **c** Primary cortical astrocytes were maintained in basal conditions (control, cont), or in Hanks’ balanced salt solution (starvation, starv) overnight. The levels of S1P were measured by liquid chromatography and mass spectrometry. The bar graph represents relative S1P levels. ***p = 0.0001 (*t* test). Results were pooled from three independent experiments. **d** A cohort of astrocytes was transfected with mApple and SK1-GFP. Two cohorts of astrocytes were transfected with mApple and a plasmid that encodes a dominant-negative form of SK1 (dnSK1) tagged to GFP. Astrocytes were maintained in Hanks’ balanced salt solution (SK1 + starv or dnSK1 + starv). A cohort of dnSK1-expressing cells was treated with 1 µM S1P (dnSK1 + starv + S1P). Astrocytes were longitudinally imaged with an automated microscope. The addition of S1P partially restored survival of dnSK1-expressing astrocytes under starvation. ***p (SK1 + starv vs dnSK1 + starv) = 0.0001, **p (dnSK1 + starv vs dnSK1 + starv + S1P) = 0.0287, p (SK1 + starv vs dnSK1 + starv + S1P) = 0.0131 (log-rank test). Fifty astrocytes per group were analyzed from two independent experiments. n.s. not significant

SK1 protects cancerous cells from apoptosis during nutrient starvation^9^. We found that a dominant-negative form of SK1 (dnSK1) was toxic to starving cancerous neuroblastoma SH-SY5Y cells^8^. Here, we showed that withdrawal of essential amino acids stimulates autophagy and upregulates SK1 in astrocytes (Fig. 1i, j). To confirm that SK1 plays a role in astrocytic survival under starvation conditions, we measured astrocytic survival during amino acid starvation. Astrocytes were transfected with (1) mApple (a survival marker) + GFP, (2) mApple + SK1-GFP, and with (3) mApple + dnSK1-GFP (a dominant-negative form of SK1). Astrocytic cohorts were starved in Hanks’ balanced salt solution, and their survival was analyzed longitudinally. We discovered that dnSK1-GFP was toxic to starving astrocytes (Fig. 2a, b). In contrast, SK1-GFP protected astrocytes from nutrient starvation, recapitulating data generated in cancerous cell lines^8, 9^.

Nutrient starvation enhances phosphorylation of SK1 in astrocytes, indicating that SK1 is activated (Fig. 1j). To confirm that activated SK1 synthesizes more S1P, we used liquid chromatography-mass spectrometry (LC-MS). One cohort of astrocytes was maintained in basal medium, and another cohort was incubated in Hanks’ balanced salt solution. Cells were processed, and S1P levels were measured. In starved astrocytes, the levels of S1P were approximately fourfold higher than in control astrocytes (Fig. 2c). To further confirm that S1P is required for astrocytic survival, we transfected astrocytes with SK1 (as control) or dnSK1 and starved these cells for 24 h. S1P was added to a cohort that expressed dnSK1. Again, dnSK1 was toxic to starving astrocytes. Risk of death curves, however, indicated that exogenous S1P partially restored survival of dnSK1-expressing astrocytes under starvation (Fig. 2d).

### SK1 shortens the half-life of an autophagy substrate in astrocytes

**Fig. 3:**
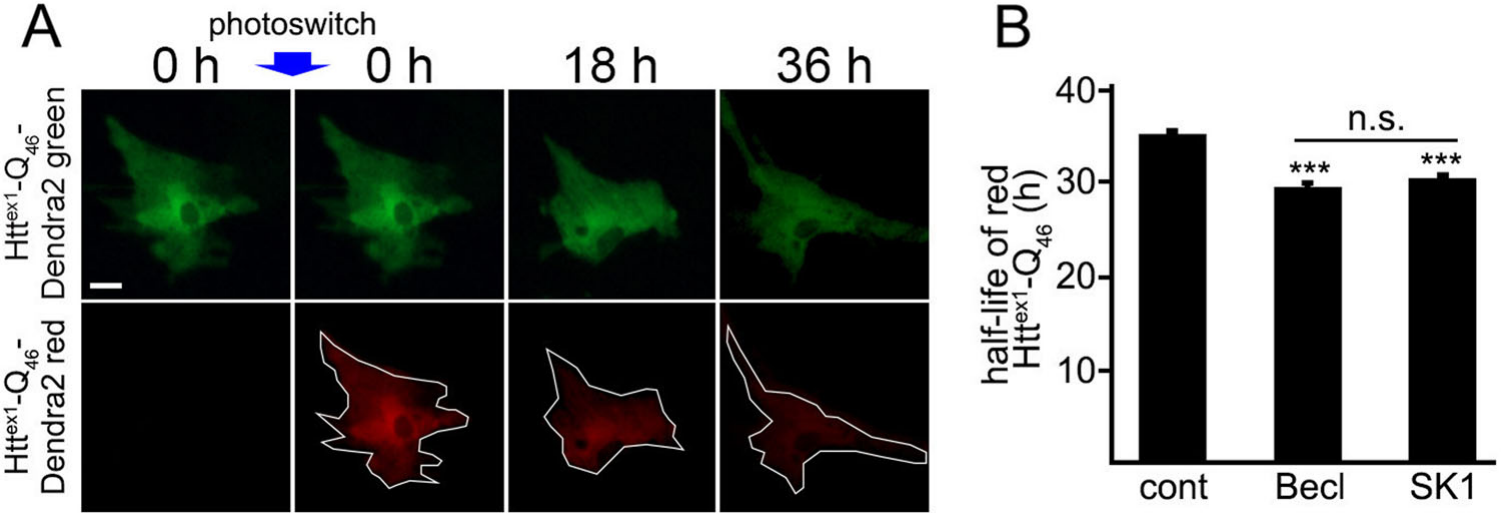
SK1 reduces the half-life of an autophagy substrate in astrocytes. **a** The photoswitchable protein Dendra2 was fused to Htt^ex1^-Q_46_ to measure autophagy flux. Brief irradiation with short-wave-length visible light causes Dendra2 to undergo an irreversible conformational change (“photoswitch”) and emit red fluorescence that can be tracked until altered Htt^ex1^-Q_46_-Dendra2 is cleared. Scale bar is 10 µm. **b** Primary astrocytes were transfected with Htt^ex1^-Q_46_-Dendra2 and an empty plasmid (control, cont), or with Htt^ex1^-Q_46_-Dendra2 and a plasmid that encodes Beclin1 (Becl), or with Htt^ex1^-Q_46_-Dendra2 and a plasmid that encodes SK1 (SK1). Beclin1 was used as a positive control. After a “photoswitch,” astrocytes were longitudinally imaged. The change in the red fluorescence intensity over time was used to calculate the half-life of Htt^ex1^-Q_46_-Dendra2. The single-cell half-life of Htt^ex1^-Q_46_-Dendra2 was significantly reduced by SK1 expression. Fifty astrocytes per group were analyzed from two independent experiments. ***p (control vs Becl) = 0.0001, ***p (control vs SK1) = 0.0001; *p* (Becl vs SK1) = 0.255 (one-way ANOVA). n.s. not significant

Although SK1 is important in astrocytic survival, the astrocytic activity of SK1 may be required for clearing an autophagy substrate, polyQ-expanded mutant huntingtin (mHtt), the protein that causes Huntington’s disease (HD). To determine if SK1 affects mHtt degradation in astrocytes, astrocytes were transfected with (1) Htt^ex1^-Q_46_-Dendra2 and an empty plasmid, or (2) Htt^ex1^-Q_46_-Dendra2 and a plasmid encoding Beclin1 (a positive control), or (3) Htt^ex1^-Q_46_-Dendra2 and a plasmid encoding SK1. Astrocytes were photoswitched, and the decay of red Dendra2 fluorescence was tracked over time (Fig. 3a). Astrocytes overexpressing SK1 and Beclin1 degraded Htt^ex1^-Q_46_-Dendra2 red fluorescence faster than control astrocytes (Fig. 3b). Our findings confirm that astrocytes are important in HD^22^. In addition to a pro-survival function of astrocytic autophagy under starvation conditions, SK1 promotes mHtt degradation. As reported^17, 23–26^, our data indicate that the S1P pathway is important in HD.

### Overexpressed SK1 is toxic to neurons

**Fig. 4:**
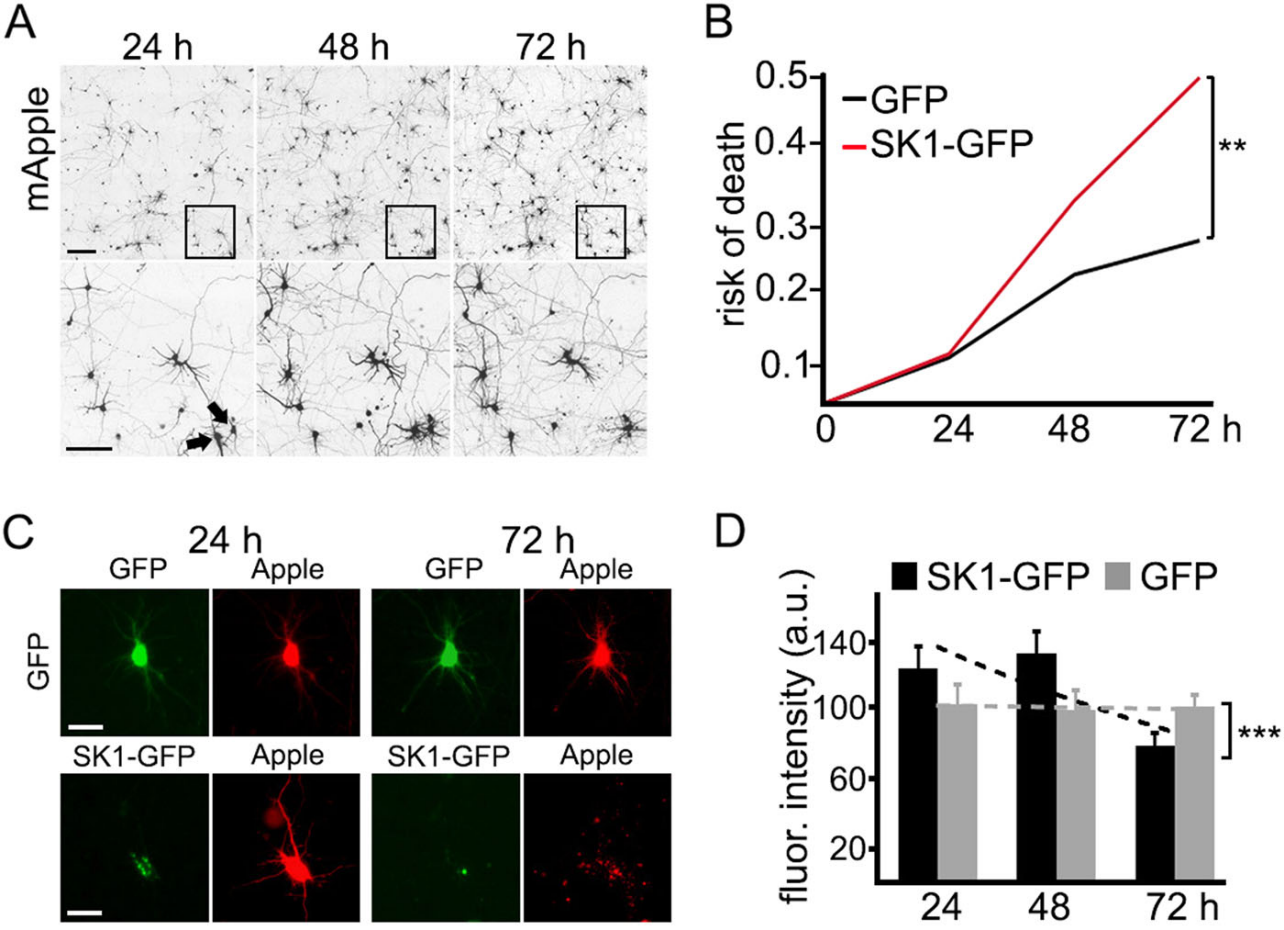
Overexpressed SK1 is neurotoxic for primary cortical neurons. **a** An example of survival analysis in neurons. Primary cortical neurons were transfected with mApple to visualize neuronal morphology. The same group of neurons was imaged 24 h after transfection and tracked over time with an automated microscope. Images collected after 24 h demonstrate the ability to return to the same field of neurons and to follow them over time. Each image in the top panels is a montage of non-overlapping images captured in one well of a 24-well plate at different time points (24, 48, and 72 h). Scale bar is 400 μm. In the bottom panel, a region from the original images is zoomed in to demonstrate longitudinal single-cell tracking. Black arrows depict two neurons that develop differently over time. The neurons on the left degenerate and disappear before 72 h after transfection, and the neuron on the right remains alive until the end of the experiment. Scale bar is 50 μm. **b** Primary cortical neurons were transfected with mApple and GFP or with mApple and SK1-GFP and tracked with an automated microscope for 72 h. Risk of death curves demonstrate that SK1-GFP expression is neurotoxic. **p (GFP vs SK1-GFP) = 0.0032 (log-rank test). Two hundred neurons were analyzed from three independent experiments. **c** Primary cortical neurons were transfected with mApple and GFP or with mApple and SK1-GFP and imaged thereafter. Note that the SK1-expressing neuron died, while the control neuron was alive until the end of the experiment. Scale bar is 10 µm. **d** Primary neurons were transfected with mApple and GFP or with mApple and SK1-GFP. Neurons were imaged 24 h after transfection, and the green fluorescence intensity was measured in each neuron. To determine the dose-dependent toxicity in cortical primary neurons that express GFP or SK1-GFP, the green fluorescence intensities in individual neurons were correlated with the time at which each cell died. The bar graphs represent the correlate of average of GFP and SK1-GFP fluorescence intensities with neuronal longevity. Note that neuronal survival is not correlated with the expression of GFP. The graph bar contains the linear correlation slopes of GFP- and SK1-GFP-expressing neurons. The SK1-GFP fluorescence intensity of each neuron was correlated with the cell's risk of death. The SK1-GFP intensity is correlated with a higher risk of death. *m* (GFP) = −0.986; *m* (SK1-GFP) = −24.864. ***p = 0.0001 (*t* test). Two hundred neurons were analyzed from three independent experiments

We and others demonstrated that substituted benzoxazines promote autophagy in neurons and enhance degradation of misfolded proteins^15, 27^. We also showed that SK1 promotes neuronal autophagy^17^. Here, we show neuroprotective benzoxazines (e.g., 10-NCP) upregulate SK1 (Fig. 1c), indicating that SK1-associated autophagy is neuroprotective. While analyzing SK1-associated autophagy in cultured neurons, we noticed that many neurons overexpressing SK1 retracted their neurites (Fig. 4a). To determine if overexpressed SK1 might be paradoxically neurotoxic for neurons, we transfected cortical neurons with mApple and GFP or with mApple and SK1-GFP and measured neuronal survival. Remarkably, SK1-GFP is neurotoxic to primary neurons (Fig. 4a–c).

Since SK1 regulates neuroprotective autophagy in neurons, and 10-NCP and FPZ activate SK1 and are neuroprotective, we questioned if neurotoxicity associated with high expression levels of SK1-GFP obscures a potential SK1-GFP-associated neuroprotection at lower SK1-GFP expression levels. The fluorescence intensity of an expressed protein (SK1-GFP) is proportional to the amount of expressed protein^23, 28–30^. By analyzing SK1-GFP levels in individual neurons, we can determine an effect of a dose-dependent toxicity on survival in a neuronal cohort. GFP levels did not correlate with a time when a neuron would die^28^. However, the levels of SK1-GFP strongly correlated with neuronal death: higher doses of SK1-GFP were more toxic (Fig. 4d). Unlike in astrocytes, overexpressed SK1 is toxic in neurons. Intriguingly, endogenous SK1 regulates cytoprotective autophagy in neurons^17^.

### Localization of SK1-GFP puncta predicts neuronal death

Activation of endogenous SK1 by 10-NCP and ectopically overexpressed SK1 results in different outcomes. One possible explanation for this seemingly paradoxical phenomenon is that, in response to 10-NCP, SK1’s enhanced activity is compartmentalized, whereas overexpressed SK1 elevates S1P levels throughout the cytosol.

Differences in S1P signaling in specific compartments in highly polarized neurons may be important. For example, caspase-3 signaling in neurons depends on the compartment. Caspase-3, when activated in distal neurites, is important for regulating spine density. Under disease conditions, caspase-3 can be overactive and act closer to the neuronal nucleus, leading to apoptosis^31^.

**Fig. 5:**
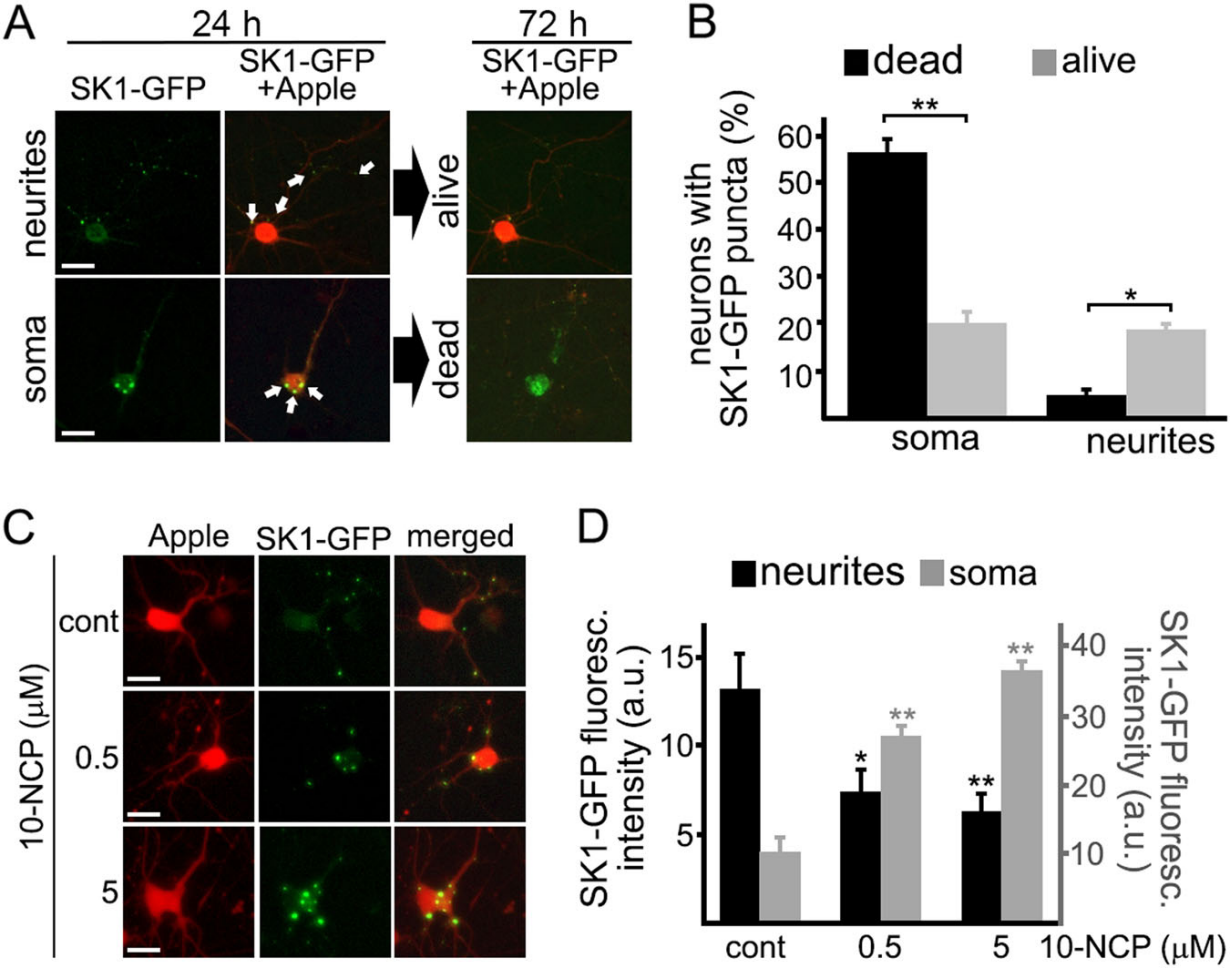
Localization of SK1-GFP puncta predicts neuronal death. **a** Cortical primary neurons were transfected with mApple and SK1-GFP, and imaged. Images demonstrate two representative neurons with the first neuron containing SK1-GFP puncta in its neurites (top panel, scale bar is 20 µm) and the second neuron containing SK1-GFP puncta in the soma (bottom panel, scale bar is 10 µm). Note that the second neuron died, whereas the first neuron remained alive. **b** Cortical primary neurons were transfected with mApple and SK1-GFP, and imaged. Bar graphs represents the percentage of neurons containing SK1-GFP puncta in the soma or in neurites 24 h after transfection, which eventually died or remained alive by the end of the experiment (72 h). **p SK1-GFP in the soma (dead vs alive) = 0.0032, *p SK1-GFP in neurites (dead vs alive) = 0.0018 (*t* test). One hundred neurons were analyzed from two independent experiments. **c** Primary cortical neurons were transfected with mApple and SK1-GFP. Twenty-four hours after transfection, cells were treated with a vehicle or with 0.5 µM 10-NCP, or with 5 µM 10-NCP for 4 h. Neurons were then imaged. Scale bar is 10 µm. **d** Single-neuron analyses from (**c**). SK1-GFP fluorescence intensity was analyzed in neurites (black bars) and in the neuronal soma (gray bars). **p (cont vs 0.5) = 0.0144 and *p (cont vs 5) = 0.0041 in neurites. **p (cont vs 0.5) = 0.0144 and *p (cont vs 5) = 0.0041 in the soma. *p* (0.5 vs 5) = 0.848 (one-way ANOVA). Fifty neurons were analyzed from two independent experiments. A.u. arbitrary units, n.s. not significant

We showed that SK1 relocalizes to endosomes and amphisomes in neurons treated with 10-NCP^17^. Here, we show 10-NCP activates SK1 (Fig. 1c), suggesting we can use membrane-bound SK1 to indicate its activity. Automated microscopy and longitudinal analysis allow us, in living neurons, to simultaneously analyze features such as protein localization in individual neurons and predict neuronal survival or death. We hypothesized that localization of SK1-GFP-positive puncta, the membrane-bound form of SK1, predicts neuronal fate. We expressed mApple and SK1-GFP in neurons, imaged them over time, and applied statistical approaches. SK1-GFP puncta localized to the soma were associated with neuronal death. Neurons that contained SK1-GFP puncta in their neurites survived significantly better (Fig. 5a, b). Our data suggest a complex relationship between the localization of SK1 activity and neuronal survival. In addition, higher doses of 10-NCP promoted formation of SK1-GFP puncta in the soma and increased the size of SK1-GFP puncta, which could reflect enhanced retrograde transport of SK1-GFP-positive structures to the soma or their fusion^10, 11^ (Fig. 5c, d).

### Overexpressed SK1 induces excessive autophagy in the soma

Autophagy is protective in neurons, but under certain circumstances (e.g., excitotoxicity), neuronal autophagy is harmful^32–35^. Neurons exhibit a gradient of proteolytic capacity, with the soma being more proteolytically active^36–38^. This suggests that overactive or sustained autophagy is more damaging to the somatic compartment than to the processes.

**Fig. 6:**
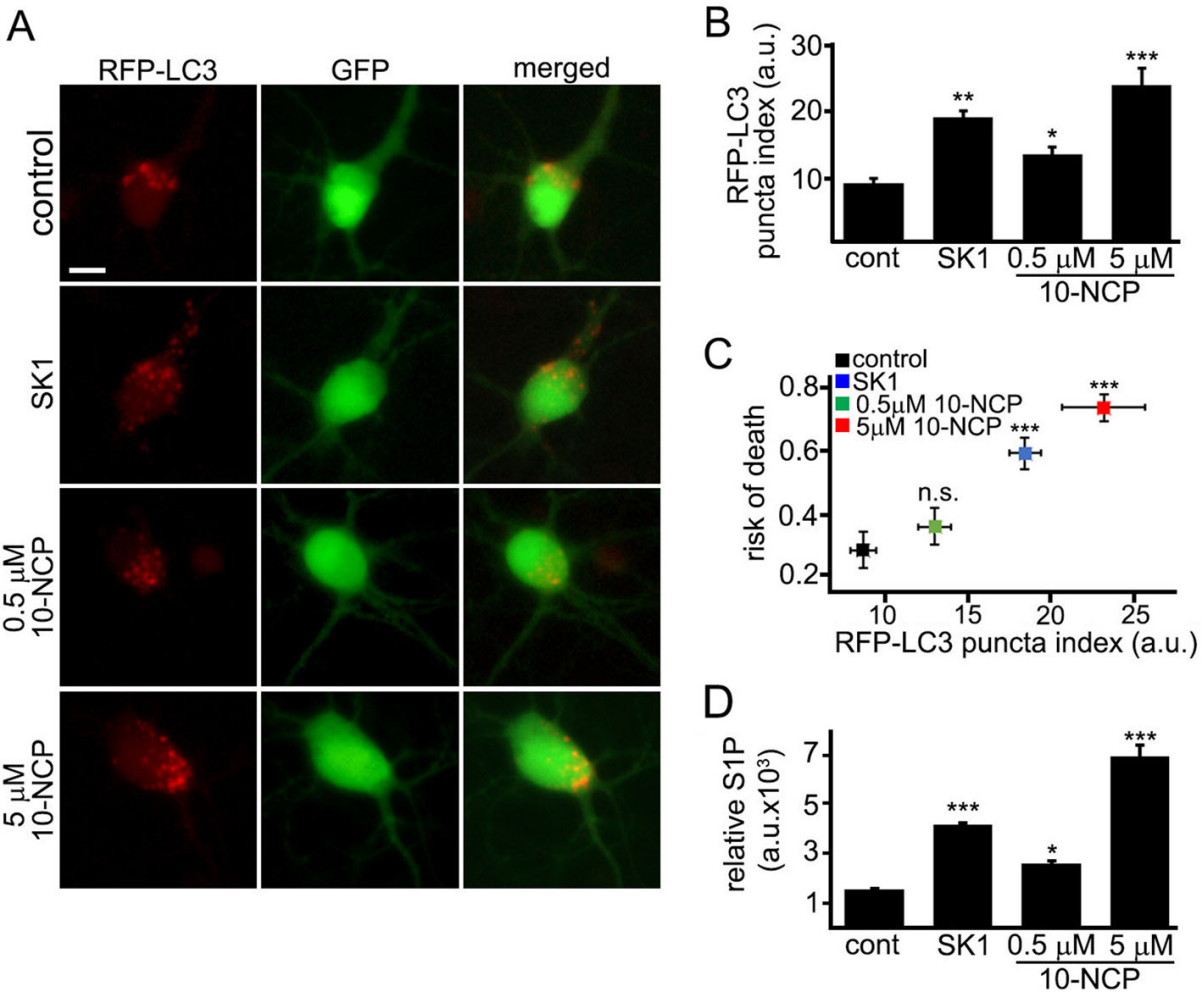
Overexpressed SK1 induces excessive autophagy in the soma. **a** Three cohorts of primary neurons were transfected with RFP-LC3, GFP, and an empty plasmid. The fourth cohort of neurons was transfected with RFP-LC3, GFP, and a plasmid that encodes SK1. The first three cohorts of transfected neurons were treated with a vehicle (control), or with 0.5 µM 10-NCP or with 5 µM 10-NCP for 4 h, respectively. Neurons were imaged 24 h after transfection. Scale bar is 20 µm. **b** Quantification of the RFP-LC3 puncta index from **a**. SK1-expressed neurons and neurons treated with 5 µM 10-NCP exhibit higher RFP-LC3 puncta index compared to control (cont). ***p (cont vs 5 µM 10-NCP) = 0.0001, **p (cont vs SK1) = 0.0019, *p (cont vs 0.5 µM 10-NCP) = 0.0342 (one-way ANOVA). Two hundred neurons were analyzed from three independent experiments. **c** The graph represents a correlation between RFP-LC3 puncta index from **b** and risk of death of four cohorts of neurons (neurons transfected with RFP-LC3, GFP, and an empty plasmid, and treated with a vehicle; neurons transfected with RFP-LC3, GFP, and a plasmid that encodes SK1; neurons transfected with RFP-LC3, GFP, and an empty plasmid, and treated with 0.5 µM 10-NCP for 4 h; and neurons transfected with RFP-LC3, GFP, and an empty plasmid, and treated with 5 µM 10-NCP for 4 h. ***p (cont vs 5 µM 10-NCP) = 0.0001, ***p (cont vs SK1) = 0.0001; *p* (cont vs 0.5 µM 10-NCP) = 0.352 (log-rank test). Two hundred neurons were analyzed from three independent experiments. **d** Four cohorts of primary cortical neurons were cultured. Three cohorts of neurons were nucleofected with GFP. The first cohort was treated with a vehicle (control, cont). The second and the third cohorts were treated with 0.5 or 5 µM 10-NCP for 4 h. The fourth cohort was nucleofected with SK1-GFP (SK1). Neurons were plated and maintained for 36 h. Neurons were then collected, and the levels of S1P were measured by liquid chromatography and mass spectrometry. The bar graph represents the S1P levels normalized with S1P content in the control group. ***p (cont vs SK1) = 0.0001, *p (cont vs 0.5 µM 10-NCP) = 0.0274, ***p (cont vs 5 µM 10-NCP) = 0.0012 (one-way ANOVA). Results were pooled from three independent experiments. A.u. arbitrary units, n.s. not significant

To assess autophagy in the soma, four neuronal cohorts were analyzed: (1) neurons transfected with RFP-LC3, GFP, and an empty plasmid, and treated with a vehicle; (2) neurons transfected with RFP-LC3, GFP, and a plasmid that encodes SK1; (3) neurons transfected with RFP-LC3, GFP, and an empty plasmid, and treated with a neuroprotective dose of 10-NCP (0.5 µM); (4) neurons transfected with RFP-LC3, GFP, and an empty plasmid, and treated with a high neurotoxic dose of 10-NCP (5 µM). The RFP-LC3-positive puncta were abundant in SK1-expressing neurons and neurons exposed to high doses of 10-NCP (Fig. 6a, b). Neurons treated with a neuroprotective dose of 10-NCP contained less RFP-LC3-positive puncta in the soma, indicating lower levels of autophagy (Fig. 6a, b). To determine if high levels of autophagy predict neuronal survival, the RFP-LC3 puncta index in each cell was associated with its risk of death (Fig. 6c). More puncta in the soma correlated with higher neuronal death. Our data suggest that neurons are sensitive to excessive autophagy associated with the SK1 pathway.

10-NCP upregulates SK1 activity and induces autophagy in a dose-dependent manner in neurons. Further, SK1-expressing neurons exhibit higher autophagy than neurons treated with low doses of 10-NCP. We wondered if the levels of S1P vary among doses of 10-NCP and in neurons that overexpress SK1. Neurons were nucleofected either with GFP or with SK1-GFP. At 48 h after nucleofection, GFP-expressing neurons were treated with a vehicle or with 0.5 µM or with 5 µM 10-NCP overnight. S1P levels were analyzed by LC-MS (Fig. 6d). The neuroprotective dose of 10-NCP increased S1P levels only slightly, whereas overexpressed SK1 and a high dose of 10-NCP raised S1P levels more significantly (Fig. 6d). This suggests that, in addition to a spatial regulation of SK1, there is a threshold S1P level that switches from a neuroprotective pathway to a neurotoxic one.

### Overexpressed SK1 induces DNA damage in neurons but not in astrocytes

Elevating S1P levels in the neuronal nucleus promotes DNA damage^23^. Intriguingly, S1PL, being a cytoplasmic enzyme, nevertheless modulates DNA damage responses in cancer cells and in fibroblasts^39^. We wondered whether cytoplasmic S1P induces DNA damage in neurons and astrocytes. Three cohorts of neurons were transfected with mApple and treated with a vehicle or with a high dose of 10-NCP (5 µM) to generate S1P, or with etoposide to induce DNA damage^17, 40^. The fourth neuronal cohort was transfected with SK1-mApple (Sup. Figure 1A, B). Three cohorts of astrocytes were transfected with mApple and were treated with a vehicle or incubated in Hanks’ balanced salt solution or with etoposide. The fourth astrocytic cohort was transfected with SK1-mApple (Sup. Figure 1C, D). Neuronal and astrocytic cultures were fixed and stained with an antibody against phosphorylated histone H2A variant X (γH2A.X), a marker of DNA double-strand breaks (DSBs) (Sup. Figure 1A–D). Etoposide promoted the accumulation of γH2A.X in neurons and astrocytes. In neurons, a neurotoxic dose of 10-NCP (5 µM) and SK1 expression led to DNA damage. Surprisingly, DNA DSBs were absent in starved astrocytes and in those that express SK1. We also compared S1P levels in neurons and astrocytes and uncovered a substantial difference in these cell types: levels of S1P in astrocytes were basally ~10-fold higher than in primary neurons (Sup. Fig. 1E).

We previously demonstrated that elevating S1P levels in the neuronal nucleus alters histone H4 acetylation^23^. The SK2/S1P complex inhibits histone deacetylates (HDAC)1/2^41^. Pharmacological inhibition and knockdown of HDAC1 result in the formation of DNA DSBs^42^, which agrees with our findings^23^. Since we observe that SK1 also promotes DNA damage in neurons (Sup. Figure 1), we wondered whether cytoplasmic S1P also affects histone H4 acetylation in neurons and astrocytes. First, neurons and astrocytes were transfected with GFP or with SK1-GFP, fixed, and stained with an antibody against acetylated histone H4 (Sup. Figure 2A–D). SK1-expressing neurons exhibited lower histone H4 acetylation than control GFP-expressing cells. This is surprising because we previously showed that nuclear SK2 enhances histone H4 acetylation^23^. Histone H4 deacetylation is important in neuronal death^43, 44^. Therefore, lower histone H4 acetylation may be an indicator of unfolding neurodegeneration. Histone H4 acetylation in astrocytes was not affected by SK1-GFP (Sup. Figure 2C, D). Overall, our findings illustrate the complexity of the S1P pathway and highlight the differences between neurons and astrocytes in SK1-associated signaling and autophagy.

## Discussion

In this study, we investigated whether SK1 signaling and SK1-associated autophagy are differentially regulated between neurons and astrocytes. We demonstrate that substituted benzoxazines, which stimulate neuroprotective autophagy^15, 27^, upregulate SK1 in neurons, but not in astrocytes. Nutrient starvation induces SK1-associated autophagy in astrocytes, but not in neurons. Overexpressed SK1 is cytoprotective in astrocytes, but paradoxically toxic to neurons. SK1 localized to the neuronal soma leads to cell death; however, SK1 signaling restricted to neurites is not toxic. Overexpressed SK1 induces excessive somatic autophagy and DNA damage, whereas a moderate activation of endogenous SK1 via 10-NCP is associated with neuroprotection. Our data show that SK1 signaling is specific for cell type and compartment, and SK1-associated autophagy depends on cell type.

S1P is a second messenger involved in a number of extracellular, cytosolic, and nuclear signaling pathways^45, 46^. Sphingosine kinases (cytoplasmic SK1 and nuclear/mitochondrial SK2) catalyze the phosphorylation of sphingosine forming S1P. In non-neuronal cells, SK1 can be activated by a variety of factors, including growth factors and numerous GPCR ligands^47^. SK1 translocates to the plasma membrane and to endosomes in non-neuronal cells^8, 10, 48^. In neurons, activated SK1 is recruited to endosomes and amphisomes, where it regulates endocytosis^49^ and autophagy^17, 50^. In the nucleus, S1P regulates gene expression^23, 40^. S1P phosphatases remove phosphate groups from S1P, generating a pro-apoptotic lipid sphingosine^6^. S1PL irreversibly degrades S1P to phosphoethanolamine and hexadecenal molecules^7^. Interestingly, phosphoethanolamine itself positively regulates autophagy^13^. Nevertheless, genetic downregulation and pharmacological inhibition of S1PL protects against ischemia, infection, and inflammatory insults^39, 51, 52^. Inhibiting S1PL has been proposed as an immunomodulatory therapy for rheumatoid arthritis^53, 54^. An inhibitor of S1PL promotes muscle regeneration^55, 56^. Novartis reported that S1PL inhibitors may be possible agents for multiple sclerosis^57^. However, S1PL knock-out mice exhibit altered pre-synaptic architecture, with reduced density of pre-synaptic vesicles, downregulated pre-synaptic proteins, and cognitive deficits^58^. Therefore, the S1P pathway is a highly intricate, intertwined, and dynamic signaling cascade, targeting of which nevertheless may result in the development of neurotherapeutics.

The differences between neuronal and astrocytic autophagy are striking, but not unexpected. When energy sources are low, autophagy is upregulated in astrocytes^59^. In contrast, neurons appear not to upregulate autophagy under starvation conditions^3^, supporting our data that autophagy is specific to cell type. The same treatments have opposite autophagic responses in different cell types: under ethanol treatment, autophagy is enhanced in astrocytes, but downregulated in neurons^60^. In Alzheimer’s disease patients, glia cells contain higher levels of the transcription factor TFEB, which regulates lysosomal biogenesis and autophagy, in the nucleus than neurons. Thus, autophagy might be differentially regulated in glia cells and neurons^61^. In amyotrophic lateral sclerosis patients, p62 often accumulates in astrocytes, but not as much in oligodendrocytes and neurons^62^. Here, we measured S1P levels between neurons and astrocytes and discovered that S1P levels in astrocytes were basally ~10-fold higher than in neurons (Sup. Fig. 1Е), suggesting a complex relationship between S1P levels, S1P localization, and autophagic pathways. Therefore, studying differences of autophagy regulation in different types of brain cells will help understand how they contribute to neurodegenerative diseases.

Autophagic mechanisms differ between neuronal compartments^3, 63–66^. Axonal autophagy delivers mature autophagosomes with cargo from the distal axon to the soma^67^. The soma also contains locally generated autophagosomes. Active lysosomes are more enriched in the soma than the axon, establishing a proteolytic gradient within the neuron^3^. Autophagy is active in pre-synaptic and post-synaptic terminals, where it regulates synaptic plasticity^68, 69^. These examples illustrate how complex autophagy is in neurons and that studying neurodegeneration in non-neuronal or even in cancerous neuron-like cells may be misleading.

Autophagy is protective in many neurodegenerative disorders^1^. A number of studies, however, demonstrate that autophagy can play a cytotoxic role in neurons^32–35^. Recreational drugs, such as cocaine and methamphetamine, induce excessive autophagy in neurons, leading to neurotoxicity^70, 71^. We showed benzoxazines stimulate neuroprotective autophagy and upregulate SK1, but high doses of benzoxazines and overexpressed SK1 are neurotoxic. Therefore, autophagy needs to be in a dynamic balance to achieve neuronal homeostasis.

We showed that SK1 relocalizes to endosomes and amphisomes in primary neurons treated with an autophagy inducer 10-NCP^15, 17^ and that 10-NCP activates SK1 (Fig. 1c), indicating that membrane-bound SK1 is active in neurons. Then we used an automated microscopy and single-neuron analysis platform to study how the localization of SK1-positive puncta affects survival under basal conditions. The localization of active SK1 is a predictor of neurodegeneration. Two hypotheses describe mechanisms by which elevated SK1 causes neurodegeneration. In one, similarly to caspase-3^31^, SK1 is important for maintaining neuronal homeostasis when activated in neuronal processes. Under pathogenic conditions, SK1 is overactive and acts closer to the soma, resulting in neurodegeneration. In the other, overactive SK1 places demands on homeostasis systems (e.g., autophagy) that exceed their capacity. The two hypotheses are not mutually exclusive and may most likely share some SK1-dependent mechanisms. A sensitive yet specific antibody that reliably detects phospho-SK1 in fixed cells or a fluorescent substrate for measuring the activity of SK1 in living cells would help shed light on the spatio-temporal mechanisms of the SK1 pathway in neurons.

In our study, we showed that SK1 and SK1-associated autophagy are regulated differently in two symbiotic brain cells—astrocytes and neurons. Importantly, SK1-dependent autophagy appears to play distinct roles within the neuron in the soma and neuronal processes. Therefore, understanding the regulation of autophagy in different brain cell types and in neuronal compartments may be critical for evaluating a potential therapeutic target for neurodegenerative diseases.

## Materials and methods

### Chemicals and plasmids

10-NCP (10-(4′-(*N*-diethylamino)butyl)-2-chlorophenoxazine) was from EMD Millipore (Darmstadt, Germany). Fluphenazine (FPZ) was from Sigma (San Luis, MO). Rabbit antibodies against SK1 and phospho-SK1 (Ser225) were custom made by Yenzym Antibodies, LLC (1:1000; South San Francisco, CA). Antibodies against β-actin were from Cell Signaling (clone 8H10D10, #3700, 1:2000; Danvers, MA). Antibodies against LC3 were from MBL (#PD014, 1:1000; Woburn, MA). Antibodies against rabbit IgG(H + L) conjugated with horseradish peroxidase (#AP307P, 1:3000), and mouse IgG(H + L) conjugated with Horseradish peroxidase (HRP), (#AP308P, 1:3000) were from EMD Millipore. pGW1-GFP-SK1, a dominant-negative form of SK1 (Gly81 to Asp28; GFP-tagged), pGW1-Beclin1, and pGW1-mApple were described^17^. pGW1-SK1 was cloned from pGW1-SK1-GFP by removing the GFP tag. pGW1-Htt^ex1^-Q_46_-Dendra2 was described^18^. pGW1-Dendra2-LC3 was described^18^. pGW1-TagRFP-LC3 (referred to as RFP-LC3 in the text and in the figure legends) was described^17^. TagRFP, unlike many other red fluorescent proteins, does not aggregate^72^, which makes it ideal for tagging the LC3 protein.

### Cell cultures and transfection

Cortices from rat embryos (E17 and E18) were dissected, dissociated, and plated on 24-well tissue-culture plates (4 × 10^5^ per well) coated with poly-d-lysine (BD Biosciences, San Jose, CA) as described^17, 23, 73, 74^. Primary cortical neurons were grown in Neurobasal Medium (Life Technologies, Carlsbad, CA) supplemented with B-27 (Life Technologies), GlutaMAX (Life Technologies), and penicillin–streptomycin (Life Technologies).

Primary cortical astrocytes were grown in Dulbecco’s Modified Eagle Medium (Thermo Fisher Scientific, Hampton, NH) supplemented with 10% heat-inactivated fetal bovine serum (Sigma) and penicillin–streptomycin. Primary cultures were transfected with Lipofectamine2000 (Thermo Fisher Scientific) and a total of 1–2 μg of plasmid DNA per well, as described^17, 23, 73^. In some experiments, neurons were nucleofected with the Neon Transfection System from Thermo Fisher Scientific (1000 V, 30 ms, 2 pulses).

### Western blotting

Western blotting was performed as described^75^. Cleared cellular lysates were analyzed by SDS/PAGE and proteins were transferred onto PVDF membranes by the iBlot2 system (Life Technologies). Membranes were blocked and incubated overnight with antibodies against actin, LC3, SK1, or pSK1. Membranes were washed and probed for 1 h with anti-rabbit or anti-mouse antibodies conjugated with HRP. Signals were detected using ProSignal Pico (Genesee Scientific, El Cajon, CA) on Medical X-Ray Film (Kodak, Rochester, NJ).

### Fluorescence microscopy

Cell imaging was performed using the EVOS microscopy system (Life Technologies). The microscope automatically positions the ×20 objective to the center of the first well of the 24-well tissue plate and collects fluorescence images with the RFP filter (mApple; RFP-LC3; SK1-mApple; “photoswitched” red Dendra2) and the GFP filter (GFP; SK1-GFP; dnSK1-GFP; “non-photoswitched” Dendra2; γH2AX).

### Optical pulse-chase

Studying autophagy in primary cells is technically difficult. Our inability to biochemically measure autophagy in transfected neuronal and astrocytic cultures led us to develop the optical pulse-chase technique based on Dendra2, a photoswitchable protein. Photoswitching of Dendra2-LC3 and Htt^ex1^-Q_46_-Dendra2 was performed as described^16–18^. Upon brief irradiation with short-wave visible light, Dendra2 undergoes an irreversible conformational change (“photoswitch”). The spectral properties of Dendra2 then change from that of a protein that absorbs blue light and emits green fluorescence to that of one that absorbs green light and emits red fluorescence^16^. Photoswitched Dendra2 maintains these spectral properties until the cell degrades the protein. The red fluorescence intensities from a region of interest in individual cells were measured at different time points. Fluorescence of non-photoswitched “green” molecules served as a guide for drawing the region of interest. The decays of red fluorescence were plotted against time, transformed into log values, and individual half-life (*t*_1/2_) was analyzed^16, 18^. *t*_1/2_ = (Ln(2)/*λ*), were *λ* is the decay value. The bar graph represents the average and standard error of the mean of half-life.

### Image analysis

Puncta formation and puncta indexes were analyzed as described^15, 76^. Briefly, the redistribution of SK1-GFP, RFP-LC3, and γH2AX into punctate structures was reflected by the puncta index, which is the standard deviation of the intensities measured among pixels within the cellular region of interest. Diffuse localization corresponds to a low puncta index, and punctate localization corresponds to a high puncta index.

### Survival analysis

Primary neurons were transfected with GFP and mApple or with SK1-GFP and mApple. In another experiment, primary neurons or astrocytes were transfected with mApple (a morphology and viability marker) and SK1-GFP or dnSK1-GFP. Cells were imaged every 24 h thereafter.

We used automated microscopy and longitudinal analysis to analyze cell survival. This method allows us to track large cellular cohorts and to sensitively measure their survival with the statistical analyses used in clinical medicine^15, 17, 23^. For tracking the same group of cells over time, an image of the fiduciary field on the plate was collected at the first time point and used as a reference image. Each time, the same plate was imaged, the fiduciary image was aligned with the reference image. We used GFP or mApple fluorescence intensities as cell morphology and survival markers. Loss of the green GFP or red mApple fluorescence is a sensitive marker of neuronal death^28, 29^. Neurons that died during the imaging interval were assigned a survival time. These events were transformed into log values and plotted in risk of death curves and analyzed for statistical significance (log-rank test). JMP software (SAS Institute, Houston, TX) was used to analyze data and generate curves^18, 77^.

### Metabolic profiling by liquid chromatography and mass spectrometry

S1P levels were measured by LC and MS, as described^23^. Agilent 1290 HPLC coupled with the 6495 triple quadrupole mass spectrometer was used to measure S1P. The compound was separated using a HILIC column; C17-S1P was used as an internal standard. Given that nucleofection efficiency was ~40% in SK1-GFP-expressing neurons, the relative levels of S1P were normalized to 100% with the formula S1P_(normalized SK1-GFP)_ = [S1P_(control)_ + ((S1P_(SK1-GFP)_ − S1P_(control)_) × 0.4)]. Data were then statistically analyzed by the one-way analysis of variance (ANOVA) test.

### Statistical analysis

For longitudinal survival analysis, neurons or astrocytes that died during the imaging interval were assigned a survival time (the period between transfection and their disappearance from an image). These event times were used to generate exponential cumulative survival curves in JMP statistical software. Survival curves describe the risk of death for single cells in the group being longitudinally imaged. To determine differences in the survival curves, they were then analyzed for statistical significance by the log-rank test as described^77^.

To compare differences across two groups, the groups were analyzed with Student’s *t* test. Differences across multiple groups were analyzed with one-way ANOVA.

### Ethics statement

Rats were maintained in accordance with guidelines and regulations of the University of Texas McGovern Medical School at Houston (the protocol number #AWC-16-0081). All experimental protocols were approved by the University of Texas McGovern Medical School at Houston. The methods were carried out in accordance with the approved guidelines.

## Electronic supplementary material

Supplementary Info
