# Supplementary material for: Sphingosine kinase 1-associated autophagy differs between neurons and astrocytes
Source: Cell Death Dis. 2018 May 9;9(5):521. doi: 10.1038/s41419-018-0599-5 (PMC5943283; doi:10.1038/s41419-018-0599-5)
Supplement: Supplementary file 1 — Supplementary Info [file 41419_2018_599_MOESM1_ESM.docx]

**Sphingosine kinase 1-associated autophagy differs between neurons and astrocytes**

**Jose F. Moruno-Manchon^1^, Ndidi-Ese Uzor^1,5^, Chandrashekar R. Ambati^2^, Vivekananda Shetty^2^, Nagireddy Putluri^2^, Chinnaswamy Jagannath^3^, Louise D. McCullough^4,5^ and Andrey S. Tsvetkov^1,5,6^**

^1^Department of Neurobiology and Anatomy, University of Texas McGovern Medical School, Houston, TX 77030;

^2^Department of Molecular and Cellular Biology, Baylor College of Medicine, Houston, TX, 77030;

^3^Department of Pathology and Laboratory Medicine, University of Texas McGovern Medical School, Houston, TX, 77030;

^4^Department of Neurology, the University of Texas McGovern Medical School at Houston, TX, 77030;

^5^The University of Texas Graduate School of Biomedical Sciences, Houston, TX 77030;

^6^UT Health Consortium on Aging, the University of Texas McGovern Medical School, Houston, TX 77030

**Correspondence to**:

Andrey S. Tsvetkov, PhD.

Department of Neurobiology and Anatomy

The University of Texas McGovern Medical School at Houston

6431 Fannin St., MSB 7.258

Houston, TX 77030

E-mail: [andrey.s.tsvetkov@uth.tmc.edu](mailto:andrey.s.tsvetkov@uth.tmc.edu)

**Supplementary Materials and Methods**

*Chemicals and plasmids*. Etoposide was from Selleck Chemicals. Antibodies against γH2AX (clone JBW301, #05-636, 1:1000) were from EMD Millipore; antibodies against histone H4 (acetyl K5+K8+K12+K16) were from Abcam (#ab177790, 1:1000). Alexa 488 secondary antibodies were from Life Technologies (#A11001; 1:1000). Hoechst dye was from Santa Cruz Biotechnology (#sc-394039).

*Immunocytochemistry*. Cultured neurons were fixed with 4% formaldehyde for 15 min at room temperature, then permeabilized in PBS containing 0.1% Triton X-100, and blocked with 1% bovine serum albumin in PBS for 1 hour at room temperature. Cells were incubated with a primary antibody diluted in blocking buffer at 4° C overnight. Cells were then washed with PBS, incubated with secondary antibodies in blocking buffer for 1 hour at room temperature, and washed three times with PBS.

**Supplementary Figure 1**

**
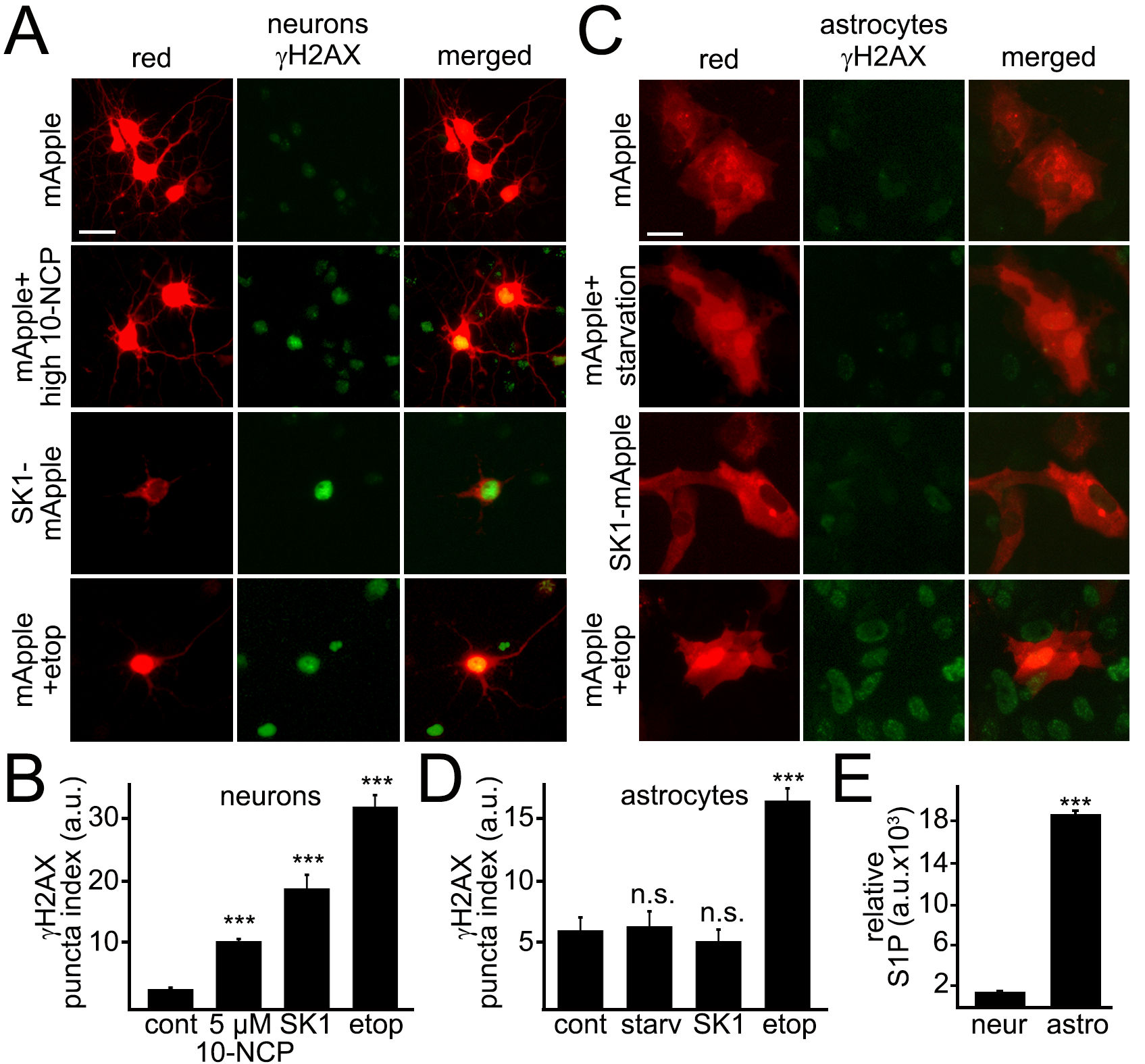
**

**Supplementary Figure 1. Overexpressed SK1 promotes the formation of DNA DSBs in neurons but not in astrocytes**. *(A)* The first cohort of primary cortical neurons was transfected with mApple and treated with a vehicle (control, mApple). The second cohort of neurons was transfected with mApple and treated with 5 µM 10-NCP (mApple + high 10-NCP). The third cohort was transfected with SK1-mApple (SK1-mApple). The fourth cohort was transfected with mApple and treated with 5 µM etoposide (mApple + etop) overnight. Neurons were fixed and stained with antibodies against a marker of DNA DSBs, phosphorylated histone H2A variant X, (γH2A.X) (green), and imaged. Scale bar is 10 µm. *(B)* The quantification of γH2A.X puncta index indicates that neurotoxic doses of 10-NCP and overexpressed SK1 damage DNA. ***p (cont vs 5 µM 10-NCP)=0.0001, ***p (cont vs SK1)=0.0001, ***p (cont vs etop)=0.0001 (one-way ANOVA). a.u., arbitrary units. Three hundred neurons were analyzed from three independent experiments. *(C)* The first cohort of primary cortical astrocytes was transfected with mApple and treated with a vehicle (control, mApple). The second cohort of astrocytes was transfected with mApple and maintained in Hanks’s balanced salt solution (starvation, starv). The third cohort was transfected with SK1-mApple (SK1-mApple). The fourth cohort was transfected with mApple and treated with 5 µM etoposide (mApple + etop) overnight. Cells were fixed and stained with antibodies against γH2A.X (green), and imaged. Scale bar is 20 µm. *(D)* The quantification of γH2A.X puncta index indicates that overexpressed SK1 does not induce DNA DSBs in primary astrocytes. ***p (cont vs etop)=0.0001; n.s., not significant, p (cont vs starv)=0.849, p (cont vs SK1)=0.5443 (one-way ANOVA). A.u., arbitrary units. One hundred fifty astrocytes were analyzed from three independent experiments. *(E)* Primary cortical neurons (neur) and astrocytes (astro) were plated, cultured and maintained in basal conditions. Cells were collected, and the levels of S1P were measured by liquid chromatography and mass spectrometry. The bar graph represents relative S1P levels. ***p =0.0001 (t-test). Results were pooled from three independent experiments.

**Supplementary Figure 2**

**
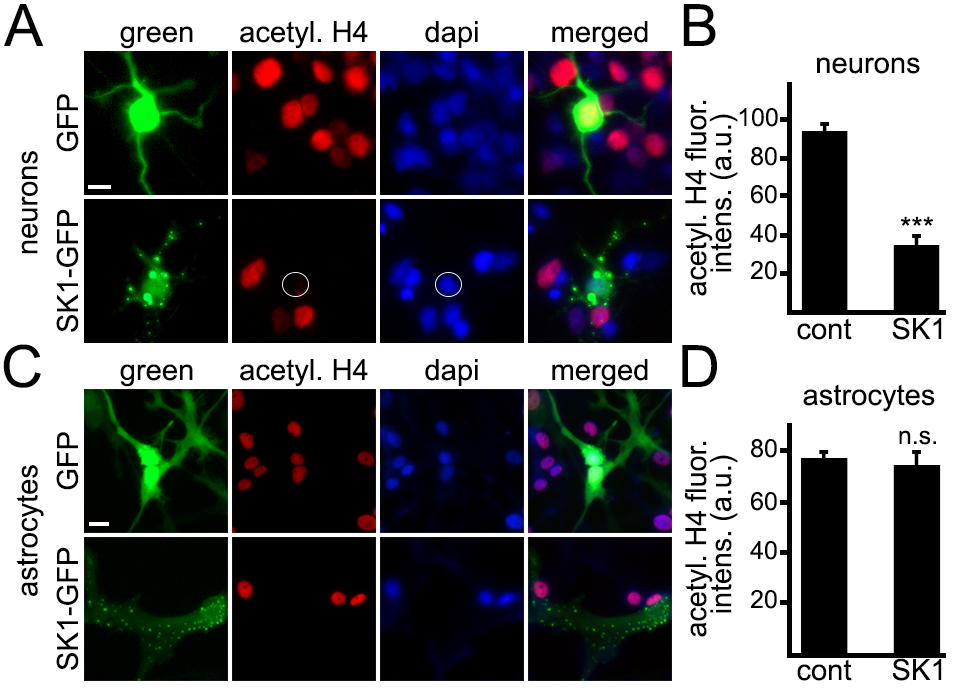
**

**Supplementary Figure 2. Overexpressed SK1 downregulates histone acetylation in neurons but not in astrocytes.** *(A)* Primary cortical neurons were transfected with GFP (control) or with SK1-GFP. 24 h after transfection, neurons were fixed and stained with antibodies against acetylated histone H4 (acetyl. H4, red), stained with Hoechst dye (DAPI, blue), and imaged. Scale bar is 5 µm. *(B)* The bar graph shows that neurons that express SK1-GFP (SK1) exhibit less fluorescence intensity of acetylated H4 than control (cont) neurons. ***p=0.0001, (t-student). A.u., arbitrary units. Two hundred neurons were analyzed from three independent experiments. *(C)* Primary astrocytes were transfected with GFP (control) or with SK1-GFP. 24 h after transfection, astrocytes were fixed and stained with antibodies against acetylated histone H4 (acetyl. H4, red), stained with Hoechst dye (dapi, blue), and imaged. Scale bar is 5 µm. *(D)* The quantification of acetylated H4 fluorescence intensity indicates that expression of SK1-GFP does not interfere with H4 acetylation in astrocytes. N.s. not significant p=0.5767, (t-student). A.u., arbitrary units. Two hundred cells were analyzed from three independent experiments.
